# Sex Differences in Estradiol Secretion by Trigeminal Brainstem Neurons

**DOI:** 10.3389/fnint.2019.00003

**Published:** 2019-02-12

**Authors:** David A. Bereiter, Randall Thompson, Mostafeezur Rahman

**Affiliations:** Department of Diagnostic and Biological Sciences, University of Minnesota School of Dentistry, Minneapolis, MN, United States

**Keywords:** aromatase, animal models, nociception, temporomandibular disorders, trigeminal nucleus caudalis

## Abstract

Estrogen status is a significant risk factor in the development of temporomandibular joint disorders (TMD). Classically, estrogen status is thought to derive mainly from ovarian sources; however, it is well known that estradiol (E2) also is synthesized by neurons in the brain. This study tested the hypothesis that E2 is produced by neurons in trigeminal subnucleus caudalis (Vc), the principal site of termination for sensory afferents that supply the temporomandibular joint (TMJ), to modify evoked responses in a model of TMJ nociception in male and female rats. Intra-TMJ injection of the small fiber excitant, allyl isothiocyanate (AIC), increased the levels of E2 collected from microdialysis probes sites at Vc of ovariectomized (OvX) female rats, ipsilateral to the stimulus, whereas males displayed no change. Dialysate levels of E2 collected from probe sites in the contralateral Vc or cerebellum in OvX rats were not affected by TMJ stimulation. Reverse dialysis of anastrozole, an aromatase (ARO) inhibitor, via the probe reduced perfusate levels of E2 in Vc. Systemic administration of letrozole, a non-steroid ARO inhibitor, for 4 days prevented TMJ-evoked increases in masseter muscle electromyography (MMemg) activity. ARO-positive neurons were distributed mainly in superficial laminae (I-III) at Vc and cell counts revealed no significant difference between OvX and male rats. Intra-TMJ injection of AIC revealed similar numbers of ARO/Fos dual-labeled neurons in OvX and male rats. By contrast, the percentage of ARO neurons co-labeled for glutamic acid decarboxylase (GAD), the biosynthetic enzyme for GABA, was greater in OvX (35%) than male rats (14%). Few ARO-positive neurons were co-labeled for estrogen receptor alpha. These data indicate that E2 is secreted continuously by Vc neurons and that acute stimulation of TMJ nociceptors evokes further secretion in a sex-dependent manner. Reduced TMJ-evoked MMemg activity after ARO inhibition suggests that locally produced E2 by Vc neurons acts via paracrine mechanisms to modify TMJ nociception in female rats.

## Introduction

Temporomandibular joint disorders (TMD) are often accompanied by pain in the temporomandibular joint (TMJ) and masticatory muscles and are more prevalent in women than men (Cooper and Kleinberg, 2007; Isong et al., 2008; Visscher et al., 2015). Genetic, biological, and psychosocial factors likely contribute to the risk to develop TMD (Diatchenko et al., 2006; Maixner, 2009). Estrogen status is thought to play a key role; however, the mechanisms that underlie the relationship between estrogen status and TMD remains uncertain (Bereiter and Okamoto, 2011). Evidence that ovarian sources of estrogen contribute to the development of painful TMD is supported by findings that the prevalence of TMD is greatest during a woman’s reproductive years and diminishes after menopause (Cooper and Kleinberg, 2007; Goncalves et al., 2010), and that ongoing or evoked jaw muscle pain fluctuates over the menstrual cycle (Suenaga et al., 2001; Isselee et al., 2002; LeResche et al., 2003; Sherman et al., 2005). However, it is well established that estrogens also are produced by a wide variety of non-ovarian tissues, including neurons, through the conversion of androgen precursors by the P450 enzyme aromatase (ARO) (Simpson, 2003; Cui et al., 2013). ARO-positive neurons are found in brain regions associated with affective and sensory-discriminative aspects of pain (Horvath and Wikler, 1999; Evrard and Balthazart, 2004; Biegon, 2016) including the trigeminal system (Evrard et al., 2004; Tran et al., 2017). Emerging evidence suggests that local production of estradiol (E2), the main biologically active form of estrogen, by brain neurons serves multiple functions from neuroprotection to altered behavior (Boon et al., 2010; Srivastava et al., 2011; Cui et al., 2013). The fact that E2 can act rapidly to alter neuronal excitability and synaptic activity (Woolley, 2007; Micevych and Mermelstein, 2008) implies that there must be a source of E2 that can fluctuate more rapidly than that derived from ovarian sources.

Primary sensory neurons that supply the TMJ region terminate in trigeminal subnucleus caudalis (Vc) (Shigenaga et al., 1988; Hathaway et al., 1995). Previously, we reported that the encoding properties of TMJ-responsive neurons at Vc varied over the estrous cycle (Okamoto et al., 2003) and were modified by chronic (Tashiro et al., 2007) and acute administration of E2 (Tashiro et al., 2012). These studies indicated that E2 likely acts via nuclear- and membrane-initiated receptor mechanisms (McEwen, 2001; Amandusson and Blomqvist, 2013) to alter TMJ nociceptive processing. The primary aim of this study was to determine if E2 is actively secreted by neurons in Vc and if this secretion contributes to TMJ nociception in a sex-dependent manner.

## Materials and Methods

The protocols were approved by the Institutional Animal Care and Use Committee of University of Minnesota and conformed to established guidelines set by The National Institutes of Health guide for the care and use of laboratory animals (PHS Law 99–158, revised 2002). All efforts were made to minimize the number of animals used for experiments and their suffering.

### Animals

Age-matched, adult intact and castrated males (280–410 g) and intact and ovariectomized (OvX) female rats (230–370 g, Sprague-Dawley, Harlan, Indianapolis, IN, United States) were used. Low estrogen status of OvX female rats was confirmed on the day of the experiment by the vaginal smear cytology as containing only small nucleated leukocytes. Arterial blood was taken at the end of most experiments and plasma E2 determined by ELISA (Invitrogen, Camarillo, CA, United States). Plasma E2 levels were less than 10 pg/ml in males, castrated males, and OvX females, while levels ranged from 15 to 65 pg/ml (*n* = 4) in intact females.

### Microdialysis

A total of 52 rats were used in microdialysis experiments. The majority of experiments were performed on intact adult male and untreated OvX female rats and at least 3 weeks after OvX surgery. Castrated males (*n* = 4) and intact female rats (*n* = 4) also were used to determine if gonadal sources of E2 contributed levels measured in microdialysis samples.

#### Animal Preparation

**FIGURE 1:**
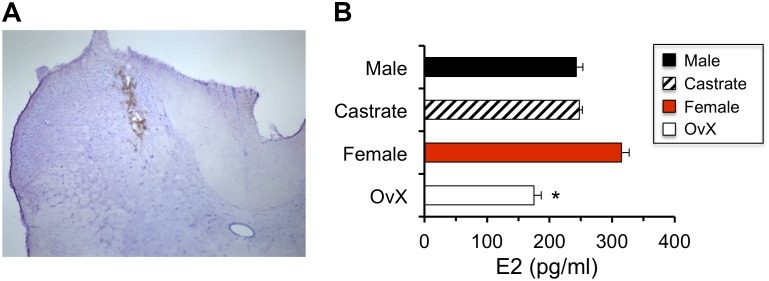
Estradiol (E2) values measured in microdialysis samples were reduced in OvX female rats. **(A)** Example of probe placement for collecting microdialysis samples in caudal Vc (coronal section; I-III and IV-V = laminae). **(B)** Effect of gonadectomy in males and females on E2 values measured in microdialysis samples; ^∗^*p* < 0.05 versus intact female.

After an initial dose of pentobarbital sodium (60 mg/kg, i.p.) a catheter was placed in the right femoral artery (blood pressure monitor) and the trachea. Rats were respired artificially and maintained with isoflurane (1.5∼2.0%) and oxygen-enriched room air. Adequate depth of anesthesia was confirmed by the loss of hindlimb withdrawal reflexes and constant mean arterial blood pressure (MAP, 90–120 mmHg) and expiratory end-tidal CO_2_ (3.5–4.5%). Body temperature was maintained at 38°C with a heating blanket and thermal control unit. Rats were placed in a stereotaxic frame and portions of the C_1_–C_2_ vertebrae were removed to expose the caudal Vc region. The atlanto-occipital membrane was cut at the level of the obex and a small portion of the pial membrane covering the brainstem was removed to allow insertion of the microdialysis probe. The microdialysis probe was directed at the Vc at approximately 10° off vertical and angled rostrally to maximize the dialyzable portion of the probe within the dorsal horn. The concentric microdialysis probe had a 1 mm membrane exposure length, 0.24 mm outer diameter, and 6 kDa cutoff (model CMA7, CMA/Microdialysis, Solna, Sweden). The probe was positioned immediately rostral to the C2 rootlets, 1–2 mm lateral to the midline and advanced ventrally (∼1 mm) until the dialysis membrane was completely below the brainstem surface (see Figure 1A). The microdialysis probe was perfused with artificial CSF (150 mM NaCl, 2.6 mM KCl, 1.3 mM CaCl_2_, 1.8 mM MgCl_2_, pH 6.5) delivered by a nanoliter pump (CMA, Model 100) at a flow rate of 2 μl/min. Dialysis samples were collected at 30 min intervals, kept on ice, and stored at -80°C for subsequent E2 analyses. An equilibration period of 60–90 min elapsed after probe placement before samples were collected for E2 determination. Probe recovery of E2 averaged ∼50% as determined from a stock solution of 300 pg/ml collected at 2 μl/min and averaged over five consecutive 30 min samples. Probe recovery of E2 across experiments remained stable at 51 ± 3% after use in 5–7 preparations.

### Experimental Designs

#### Effect of Gonadectomy

This experiment determined the relative contribution of gonadal sources of E2 to the levels recovered from dialysis samples at the Vc region. Males and females were gonadectomized at least 3 weeks prior to the experiment and results were compared to those of intact animals. Three consecutive 30 min samples were collected after a 60–90 min equilibration period following probe insertion.

#### Local Inhibition of ARO

This experiment determined if local application of the ARO inhibitor, anastrozole (Tocris), altered the levels of E2 recovered from microdialysis samples in OvX females. After the 60–90 min equilibration period, three 30 min samples were collected and then anastrozole (100 μM, 50 μl) was applied to the dorsal brainstem surface and simultaneously perfused through the microdialysis probe (2 μl/min for 30 min). Additional 30 min samples were collected for 150 min.

#### Effect of TMJ Stimulation

To determine if activation of TMJ nociceptors altered E2 levels recovered from Vc samples, the small fiber excitant, allyl isothiocyanate (AIC, 20%, 20 μl), was injected into the joint space of OvX females and intact male rats ipsilateral to the probe placement. Sampling began after a 60–90 min equilibration period followed by three 30 min prestimulus samples, followed by AIC injection and five additional 30 min samples. Mineral oil (20 μl) was injected into the TMJ as the stimulus control. Samples collected after intra-TMJ injections of AIC or mineral oil were made without prior knowledge of treatment. Probes placed in the Vc contralateral to the AIC stimulus and in the posterior superior fissure of the cerebellum 1 mm off the midline in separate groups of OvX females served as placement controls and followed the same AIC stimulus protocol as for ipsilateral Vc probe sites.

At the end of the experiment, animals were given a bolus of pentobarbital (60 mg/kg) and perfused through the heart with normal saline followed by 10% formalin. Probe sites were recovered histologically from transverse sections.

### Estradiol Assay

Microdialysis samples were collected on ice and stored at -80°C. After centrifugation E2 was measured with an ELISA Kit (Invitrogen). The limit of detection for E2 was 6 pg.

### Data Analyses

Microdialysis E2 levels were reported as pg/ml ± SEM and the average of the two samples collected immediately prior to treatment was considered as the baseline. Sample values were not corrected for probe recovery. E2 concentrations were assessed over time by two-way analysis of variance corrected for repeated measures. Individual comparisons across time and treatment were determined by Newman-Keuls after ANOVA.

### Masseter Muscle Electromyography (MMemg)

A total of 12 OvX rats were used to assess the effect of ARO inhibition on TMJ-evoked jaw muscle responses. The ARO inhibitor, letrozole (10 mg/kg, sc), was given daily for 4 days and TMJ-evoked MMemg was recorded on day 5. Letrozole was used in this series since it is reported to penetrate the blood brain barrier more readily than anastrozole (Miyajima et al., 2013). On day five animals were anesthetized with urethane (1.2 g/kg i.p.) and prepared surgically as for the microdialysis protocol (see above). Urethane was used to avoid the non-specific bursts of skeletal muscle activity that often accompanies prolonged barbiturate anesthesia (Chang, 1991). Animals were placed in a stereotaxic frame and the TMJ region was exposed for cannula implantation and ATP (0.01, 0.1, and 1 mM, 20 μl) injections. ATP was used as a stimulus since it can be injected repeatedly without causing tachyphylaxis or sensitization (Tashiro et al., 2007). A pair of wire electrodes (0.12 mm diameter, 5 mm interpolar distance) were implanted ∼1 mm into the central portion of the masseter muscle. MMemg activity was sampled at 1000 Hz, amplified (×10 k), filtered (bandwidth 300–3000 Hz), displayed and stored online for analyses. MMemg activity was recorded in OvX and OvX + letrozole groups evoked by cumulative doses of intra-TMJ injections of ATP (0.01, 0.1, and 1 mM, 20 μl, pH = 7.4) at 20 min intervals.

#### Data Analysis

EMG activity was sampled continuously for 6 min, from 3 min prior to each TMJ stimulus and for 3 min after stimulation. EMG activity was rectified and stored as 1 s bins for off-line analyses. Baseline activity was quantified as the area under the curve (AUC) for the 3 min epoch (μV-s per 3 min) sampled immediately prior to stimulation. TMJ-evoked MMemg activity was calculated as AUC post-ATP injection minus baseline. MMemg was assessed statistically by ANOVA corrected for repeated measures and individual comparisons were made by Newman-Keuls after ANOVA. The threshold dose of ATP was defined as the lowest concentration that increased AUC > 50% that evoked by PBS. Fisher’s Exact Probability test determined if the number of rats responding to the lowest concentration of ATP (>50% versus AUC to intra-TMJ injection of PBS) was different for OvX and OvX + letrozole groups.

### Aromatase Immunohistochemistry and Immunofluorescence

#### General Procedures

A total of 42 male and OvX female rats (200–350 g) were used to assess anatomical aspects of ARO staining in Vc. Rats were anesthetized with pentobarbital sodium (60–70 mg/kg, i.p.) and depth of anesthesia was determined by loss of the hindlimb withdrawal reflex. Four to seven animals were included in each treatment group. Rats were perfused through the heart with heparinized saline followed by 250 ml cold fixative (4% paraformaldehyde, 0.1 M phosphate, pH 7.4). The caudal Vc was removed and postfixed for 1–3 h. Transverse sections (50 μm) were cut on a vibratome and collected in cold 0.01 M phosphate buffered saline (PBS). *Immunohistochemistry*. Table 1 lists the primary antisera used in this study. Sections were incubated in 0.3% Triton X-100 with 5% blocking reagent (Background Sniper, Biocare Medical, Concord, CA, United States) for 1 h and then incubated with primary antibody for ARO (mouse, Abcam, ab139492) at 1:100 in PBS with 0.1% Triton X-100 overnight at 4°C. Sections were rinsed and placed sequentially in anti-mouse secondary antiserum conjugated to biotin (Sigma, B7264) at 1:500 in PBS for 1 h, rinsed in PBS, amplified in Vector ABC (PK-4000, Burlingame, CA, United States) for 1 h and developed with Vector DAB (SK-4100). Sections were rinsed (3× in PBS), and then incubated in antiserum for Fos protein (rabbit, Calbiochem, PC38) at 1:15,000 or for GAD65/67 (rabbit, Millipore, Ab1511) at 1:1000 in PBS-Triton-X containing 5% NDS overnight at 4°C. Anti-rabbit secondary conjugated to biotin (Millipore, AP182B) was applied at 1:300 for 1 h. After rinsing, Vector ABC was reapplied for 1 h and developed with Vector DAB (SK-4100). To co-label for ARO and ERα, sections were incubated in anti-serum for ARO (rabbit, Lifespan Bio, LS-B2816) at 1:1000 in PBS with 0.1% Triton X-100 overnight at 4°C and anti-rabbit secondary conjugated to biotin (Millipore, AP182B) at 1:500. Note that separate animals were used for protocols that stained for Fos protein as these animals received intra-TMJ injections of AIC (20%, 20 μl) and were allowed to survived for 2 h. Stimulus controls received intra-TMJ injections of mineral oil. *Immunofluorescence*. Free floating vibratome sections (50 μm) were blocked in PBS plus 0.1% Triton X-100 containing 5% NDS for 1 h. Protocols for double labeling consisted of incubation of primary antisera for ARO and either Fos, GAD or ERα overnight at 4°C. After rinsing, fluorescent-labeled secondary antibodies of donkey anti-rabbit Cy5 or anti-mouse or anti-goat Cy2 secondary antibodies for 60 min were added and incubated in the dark for 1 h at room temperature. Additional sections were incubated in primary antisera for ARO and NeuN (mouse, LV1825845, Millipore) to confirm neuronal staining. Sections were rinsed, placed on slides and coverslipped with ProLong Gold with or without DAPI (Life Technologies, Eugene, OR, United States). Specific staining was abolished by omission of primary antiserum and controls for dual labeling involved reversing the sequence of antibody applications. Fluorescent-labeled sections were viewed at 40× magnification on a Zeiss LSM 700 confocal microscope.

**Table 1:** Primary antibodies.

Antibody	Host	Manufacturer	Cat. #	Lot #	Dilution
ERα	Mouse	Millipore	MAB447	33505	1:100
Aromatase	Mouse	Abeam	ab139492	GR143409-11	1:100
Aromatase	Rabbit	Lifespan Bio	LS-B2816	27791	1:1000
cFos	Rabbit	Calbiochem	PC38	D00119667	1:15000
NeuN	Mouse	Millipore	MAB377	LV1825845	1:1000
GAD 65/67	Rabbit	Millipore	Abl511	701048886	1:1000

#### Data Analyses

Twenty to 25 sections per rat were collected from the caudal Vc region (-4.5 to -6.5 mm relative to the obex) and counted at 100× magnification on an Olympus B51A light microscope. Cell counts from non-fluorescent stained sections were made in superficial laminae (I-II) for ARO, Fos, GAD, and ERα without prior knowledge of treatment. Fos-positive neurons were identified as dark-stained nuclei, while ARO-positive or GAD-positive were seen as lighter stained cyotplasmic compartments surrounding dark-stained nuclei. Cell counts consisted of the average from three sections per rat within a standardized region of interest (ROI) of 160 μm^2^ positioned in the mediodorsal aspect of the superficial laminae that corresponded to a region of high density of Fos-positive neurons produced by noxious stimulation of the TMJ region (Hathaway et al., 1995). Cell counts were compared across treatment groups by two-way analysis of variance (ANOVA) and individual comparisons were made by Newman-Keuls after ANOVA.

## Results

### Microdialysis

The effect of gonadectomy on E2 values measured in microdialysis samples collected at the caudal Vc (Figure 1A) was determined in intact males (*n* = 6) and females (*n* = 4) and compared to castrated males (*n* = 4) and OvX females (*n* = 5). As seen in Figure 1B, castration had little effect on E2 values in dialysate samples from males, whereas E2 levels were reduced in OvX females by ∼45% compared to intact females (*F*_3,16_ = 3.70, *p* < 0.05). Thus, to minimize possible confounding effects of ovarian E2 on dialysate levels of E2, subsequent experiments were performed in intact males and OvX females.

**FIGURE 2:**
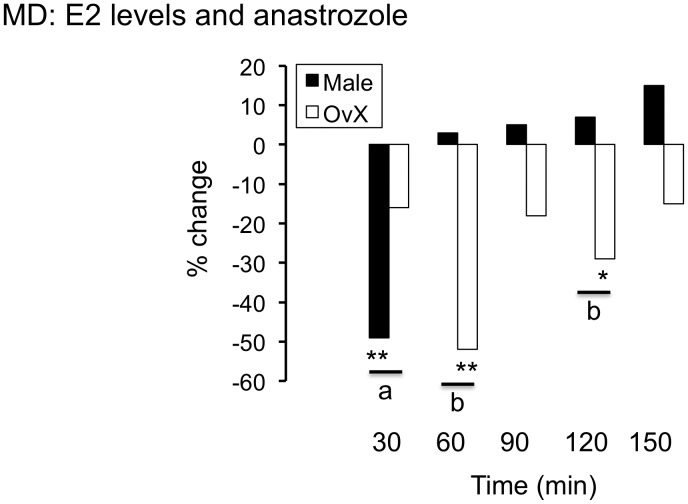
Reverse dialysis of the non-steroidal ARO inhibitor, anastrozole (100 μm, 50 μl) through probes positioned in the Vc and simultaneously topical application on the dorsal Vc surface caused a transient decrease in E2 levels in male and OvX female rats; ^∗∗^*p* < 0.01 and ^∗^*p* < 0.05 versus pre drug, *a* = *p* < 0.05 and *b* = *p* < 0.01 versus male.

The non-steroidal ARO inhibitor, anastrozole (100 μm, 50 μl), was applied simultaneously to the dorsal Vc surface at the probe site and perfused through probe during sample collection to assess the effect of local blockade of E2 biosynthesis on dialysate values. As seen in Figure 2, anastrozole caused a significant and transient decrease in dialysate E2 levels in males (*n* = 4) and OvX females (*n* = 5, *F*_5,33_ = 6.6, *p* < 0.001). Note also that the effect of anastrozole on E2 levels was more prolonged in OvX females compared to males.

**FIGURE 3:**
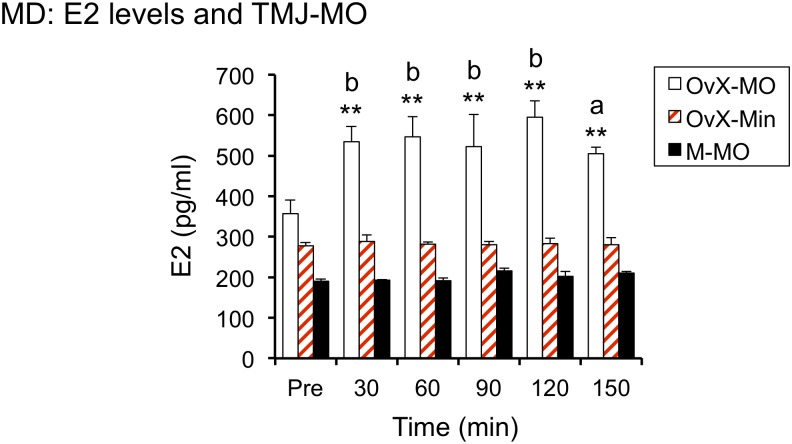
Intra-TMJ injection of 20% AIC (also known as mustard oil, MO, 20 μl) evoked a prompt increase E2 secretion in OvX but not male rats; ^∗∗^*p* < 0.01 versus pre, *a* = *p* < 0.05 and *b* = *p* < 0.01 versus Male + MO (M-MO) and OvX + Mineral oil (OvX-Min).

To determine if acute stimulation of TMJ nociceptors, ipsilateral to probe placement, was sufficient to increase dialysate E2 levels, 20% AIC was injected in OvX females (*n* = 5) and males (*n* = 4). An equal volume of mineral oil (20 μl) was injected within the TMJ joint space in OvX rats and served as stimulus controls (*n* = 7). As seen in Figure 3, AIC evoked a prompt and sustained increase in E2 in OvX females (*F*_4,18_ = 130.8, *p* < 0.001), whereas intra-TMJ injection of AIC in males or mineral oil injection in OvX females had no effect on E2 levels. Dialysis probes positioned in the Vc contralateral to the TMJ injection of AIC (*n* = 4) or in cerebellum (*n* = 3) displayed no change in E2 levels (data not shown).

### Masseter Muscle Electromyography (MMemg)

**FIGURE 4:**
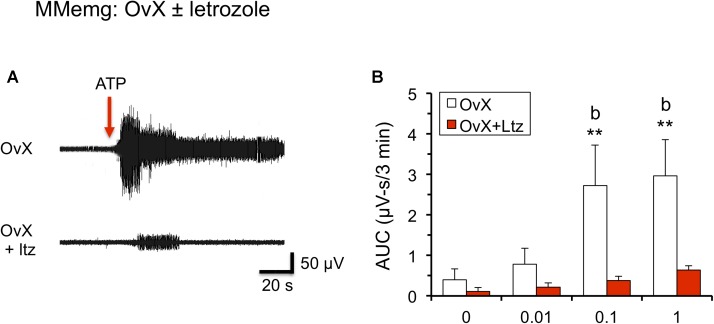
Systemic administration of letrozole (Ltz, 10 mg/kg, sc) for 4 days reduced ATP-evoked MMemg responses in OvX rats. **(A)** Example of MMemg activity evoked by intra-TMJ injection of ATP (1 mM, 20 μl) in OvX (upper panel) and OvX + Ltz rats (lower panel). **(B)** Summary of integrated MMemg activity evoked by ATP in OvX and OvX + Ltz rats. Arrow indicates the time of intra-TMJ ATP stimulation; ^∗∗^*p* < 0.01 versus vehicle, *b* = *p* < 0.01 versus OvX + letrozole (OvX + Ltz).

To determine if ARO inhibition influenced TMJ-evoked jaw muscle activity, OvX rats were treated for 4 days with letrozole (10 mg/kg/d, i.p.) and on day 5 MMemg activity was recorded ipsilateral to intra-TMJ injections of ATP. As seen in Figure 4, letrozole treated rats (*n* = 6) displayed a marked reduction in ATP-evoked MMemg activity compared to vehicle-treated OvX rats (*n* = 6, *F*_1,10_ = 8.12, *p* < 0.025).

### Immunohistochemistry

**FIGURE 5:**
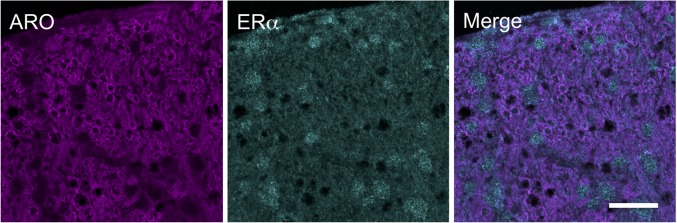
Immunofluorescent examples of ARO and ERα staining in laminae I-III of caudal Vc in an OvX rat. Note that few neurons were co-labeled. Scale = 40 μm.

**FIGURE 6:**
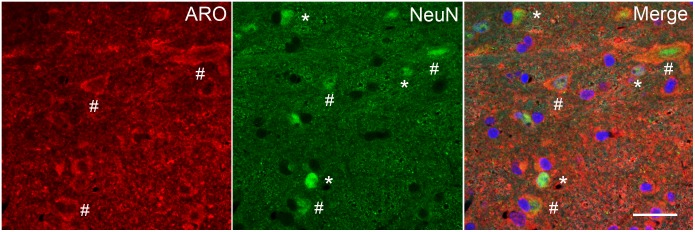
Immunofluorescent staining in laminae I-III of caudal Vc for ARO and NeuN, a neuronal marker, in an OvX rat. Since NeuN is specific for neuronal nuclei but is an incomplete marker for all neurons, the chromatin stain DAPI was added to the mounting medium in some cases. Symbols: ^∗^NeuN only; ^#^ARO/NeuN co-staining. Scale = 40 μm.

**FIGURE 7:**
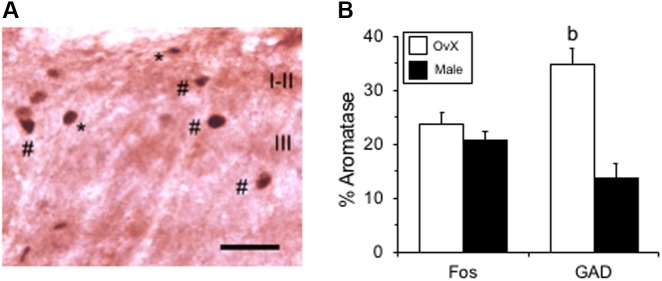
Co-labeling of ARO/Fos neurons in caudal Vc. **(A)** Example of co-labeled ARO/Fos neurons after TMJ stimulation in an OvX rat. Fos-positive neurons appeared as dark-stained nuclei (^∗^), while dual labeled ARO/Fos neurons appeared as dark nuclei surrounded by a lighter cytoplasmic compartment (#). **(B)** Summary of the percentage of ARO-positive neurons co-labeled for Fos or GAD, the biosynthetic enzyme for GABA, in male and OvX rats. *b* = *p* < 0.01 versus male. Scale bar = 30 μm.

Aromatase-positive neurons were well distributed throughout superficial laminae at the caudal Vc with only scattered cells in deeper laminae (Figure 5). Cell counts made from non-fluorescent stained sections within a 160 μm^2^ ROI in superficial laminae were similar for OvX and males (*F*_1,12_ = 0.9, *p* > 0.1). Although we did not quantify the number of ARO/ERα dual-labeled neurons a few such neurons were seen suggesting that local secretion of E2 also may serve an autoregulatory function. Neuronal phenotype was confirmed for ARO-positive cells by co-localization with NeuN (Figure 6). Since NeuN is specific for neuronal nuclei but is an incomplete marker for all neuronal nuclei (Lind et al., 2005), DAPI was included as a confirmatory nuclear stain in the mounting medium. Note that several DAPI-positive nuclei were not identified by NeuN but were surrounded by ARO-stained cytoplasm. In separate rats, sections were collected and co-labeled for ARO and Fos protein after intra-TMJ injection of AIC to determine the relationship between ARO neurons and TMJ nociceptor activation. Numerous ARO-positive neurons in superficial laminae of Vc were co-labeled for Fos after TMJ stimulation (Figure 7A). ARO/Fos positive neurons were identified by a dark-stained nucleus surrounded by a lighter stained cytoplasmic compartment. The percentage of ARO/Fos neurons within the ROI in Vc was similar in males and OvX rats (*F*_1,6_ = 1.1, *p* > 0.1); however, the percentage of ARO/GAD dual-labeled neurons was significantly greater in OvX than male rats (Figure 7B, *F*_1,5_ = 24.8, *p* < 0.005).

## Discussion

The results of this study demonstrated that E2 is produced by ARO neurons in the Vc of male and female rats. E2 is likely secreted tonically since reverse dialysis of an ARO inhibitor through the probe significantly reduced E2 levels in both males and OvX females. By contrast, acute stimulation of TMJ nociceptors by the small fiber excitant, AIC, evoked increases in E2 levels in dialysate samples from probes sites in the Vc in a sex-dependent manner. The TMJ-evoked increase in E2 in OvX females was site-specific since probes placed in the Vc contralateral to the TMJ stimulus or in the cerebellum revealed no change. Evidence that biosynthesis of E2 by ARO serves a functional role in TMJ nociception was supported by the finding that inhibition of ARO greatly reduced TMJ-evoked jaw muscle reflexes. Anatomical analyses revealed a similar number of ARO-positive neurons in superficial laminae of Vc in OvX and male rats and the percentage of dual-labeled ARO/Fos also was similar after TMJ stimulation. However, a greater percentage of ARO neurons in OvX females than males were co-labeled for GAD suggesting that TMJ nociceptors engage trigeminal brainstem circuitry differently in males and females.

It is well recognized that estrogen status plays a significant role in pain processing (Craft, 2007; Fillingim et al., 2009; Amandusson and Blomqvist, 2013; Hassan et al., 2014), including in craniofacial pain and TMD (Bereiter and Okamoto, 2011). Although classical concepts of estrogen status and pain processing have emphasized the long-term effects of E2, acting through nuclear receptors and genomic mechanisms, more recent evidence indicates that E2 also acts rapidly through non-genomic pathways to modify nociceptive responses. Acute exposure to E2 rapidly, within minutes, alters the excitability of peripheral nociceptors (Kuhn et al., 2008; Xu et al., 2008; Rowan et al., 2010; Qu et al., 2015) and dorsal horn neurons (Tashiro et al., 2012; Zhang et al., 2012b). Membrane-bound estrogen receptors (ER) on neurons are widely distributed throughout the brain (Micevych and Dominguez, 2009) and at multiple sites of somatosensory pathways (Takanami et al., 2010; Zhang et al., 2012a). Although the conversion of androgen precursors to E2 via ARO activity is a likely a source of E2 that can be rapidly modulated (Cornil and Charlier, 2010), the role of local biosynthesis of E2 in pain processing remains uncertain (Evrard, 2006).

Three lines of evidence suggested that ARO-derived E2 from the Vc contributed to TMJ nociception. First, acute stimulation of TMJ nociceptors by AIC evoked a prompt increase, by 30 min, in dialysate levels of E2. This pattern was consistent with the timing of increases in E2 seen in dialysate samples in the amygdala of the rat after arterial occlusion (Saleh et al., 2005), in hypophyseal blood samples after direct hypothalamic stimulation in the monkey (Kenealy et al., 2013) and from dialysis samples from the forebrain of songbirds (Remage-Healey et al., 2008). Similarly, samples collected from perfusion of hippocampus or spinal cord slices demonstrated a rapid increase following exogenous stimuli (Hojo et al., 2004; Zhang et al., 2012b). Second, anatomical studies revealed that ARO neurons were most numerous in superficial laminae of Vc and that ∼25% of Fos-positive neurons seen after TMJ stimulation were ARO-positive. Third, systemic administration of ARO inhibitor for 4 days completely prevented TMJ-evoked increases in MMemg activity in OvX rats. This suggested that brain pathways that mediate jaw muscle reflex activity associated with TMJ nociception rely, at least in part, on the local secretion of E2 in females. However, since the ARO inhibitor was given systemically we cannot exclude that ARO activity in other tissues including the TMJ (Yu et al., 2006) may have contributed to the reduction in TMJ-evoked MMemg activity after letrozole.

Previous studies that examined the effects of ARO activity on nociception have relied mainly on indices of evoked cutaneous reflex behavior and report mixed effects. In the quail, spinal administration of the ARO inhibitor, vorozole, caused within 1 min a reduction in behavioral responsiveness to noxious thermal stimulation (Evrard and Balthazart, 2004). Similarly, licking and biting behavior to formalin injection in the hindpaw was reduced in male and female rats 5 min after intrathecal injection of ADT, a steroid ARO inhibitor (Zhang et al., 2012a). In tumor-bearing mice, mechanical cutaneous hyperalgesia was reduced at 7 days after systemic letrozole treatment in cycling female mice but not in OvX mice (Smeester et al., 2016). By contrast, formalin-evoked behavior was increased in female ARO knockout mice (Multon et al., 2005). Systemic injection of a single dose of letrozole increased mechanical, but not thermal sensitivity, in male and OvX female rats 5 days after drug administration; however, letrozole had no effect on depolarization-evoked release of CGRP from spinal cord slices in male rats (Robarge et al., 2016). The diversity of ARO effects on behavior underscores the complexity of estrogen status involvement in nociception that likely also depends on methodological differences. Although arthralgia is a significant side effect of ARO inhibitor treatment for breast cancer, patients develop joint pain only after weeks of treatment with no report of symptoms for cutaneous hyperalgesia (Henry et al., 2008, 2014; Shi et al., 2013). Thus, there is concern about the face validity of mechanistic studies of ARO effects in animal models that do not assess joint function. The present study demonstrated for the first time that ARO inhibition significantly reduced nociceptive behavior related to jaw joint function.

A significant finding in this study was that intra-TMJ stimulation evoked an increase in E2 levels in dialysate samples at Vc of OvX females but not in males. This was unexpected given that the number of ARO neurons in superficial laminae at Vc was similar for OvX and male rats. This was consistent with recent reports obtained by a different method, i.e., immunostaining for β-galactosidase in an ARO reporter mouse that also found similar numbers of ARO neurons in Vc of male and female mice (Tran et al., 2017). The present study found that the percentage of ARO/GAD dual-labeled neurons was greater in OvX than males suggesting that many of these cells were interneurons. Tran et al. (2017) also concluded that ARO/GABA cells in Vc were interneurons; however, they did not report sex differences. A similar number of Fos-positive neurons in Vc were produced in OvX and male rats after intra-TMJ injection of AIC consistent with previous studies in which Fos production was similar for male and diestrous females (Bereiter, 2001) and for males and OvX rats given low maintenance doses of E2 (Chang et al., 2012). There are several possible explanations for observing increased TMJ-evoked secretion of E2 in OvX females, but not in males. First, the AIC stimulus may not be adequate to increase local E2 levels in Vc of males, despite the similar number of ARO and Fos-positive in OvX and males. It is possible that cell counts may not be the most critical variable, since ARO activity may be greater in females than males. Although measurable levels of prolactin are found in medullary tissue samples of male and female rats (DeVito, 1988), prolactin sensitizes Trpa1 channel activity only in female, and not male, sensory neurons (Patil et al., 2013). Second, locally secreted E2 may be metabolized differently in male and female brains. Since the ELISA assay measures only free E2, it is possible that evoked E2 is conjugated differently in male and female brains. The circulating level of sex hormone binding globulin in plasma is different in males and females (von Schoultz and Carlstrom, 1989); however, it is not known if sex differences in E2 conjugation also exist in brain tissues. Third, brain trauma, which necessarily includes the insertion of a microdialysis probe, induces the activation of astrocytes and increased expression of ARO mRNA and protein (Peterson et al., 2001). It is possible that E2 contributions from activated glia after brain injury may influence the magnitude of E2 levels evoked by a second external stimulus differently in males and females. Indeed, unilateral spinal cord injury in female rats caused a sustained increase in ARO expression and protein levels, while ARO inhibition caused a bilateral enhancement of mechanical allodynia (Ghorbanpoor et al., 2014) suggesting that local E2 secretion serves an anti-hyperalgesic function in chronic pain. It is interesting to note that in other brain areas that acute inhibition of ARO activity prevents long-term potentiation in females, but not males (Vierk et al., 2012; Bender et al., 2017) and suppresses status epilepticus in both sexes (Sato and Woolley, 2016) suggesting that ARO activity is associated with increased neuronal excitability. Previously we reported that estrogen status may gate the magnitude of GABAergic influence on TMJ-responsive neurons in Vc (Tashiro et al., 2014). Although those and the present results are consistent with the notion that sex-related differences in brain-derived E2 secretion, possibly acting through GABAergic mechanisms, play a significant role in TMJ nociception, it is not known whether ARO inhibitors enhance or reduce TMJ-evoked nociceptive behavior in a chronic animal model for TMJ nociception.

## Author Contributions

DB analyzed the data and wrote the text of the manuscript. RT and MR collected and analyzed the data and edited the text.

## Conflict of Interest Statement

The authors declare that the research was conducted in the absence of any commercial or financial relationships that could be construed as a potential conflict of interest.
